# Split-Course Adaptive Radioimmunotherapy for Oligometastatic Non-Small Cell Lung Cancer (SiCARIO): A Case Report

**DOI:** 10.7759/cureus.68868

**Published:** 2024-09-07

**Authors:** Kyra N McComas, Sarah E Masick, Adam D Yock, Ryan M Whitaker

**Affiliations:** 1 Department of Radiation Oncology, Vanderbilt University Medical Center, Nashville, USA

**Keywords:** ct-guided adaptive radiotherapy, metastatic non-small cell lung cancer, online adaptive radiotherapy (art), radioimmunotherapy, sbrt (stereotactic body radiotherapy)

## Abstract

Current treatment paradigms for oligometastatic non-small cell lung cancer (NSCLC) utilize systemic chemotherapy alone or in combination with immune checkpoint inhibitors (ICIs). The addition of ICIs in NSCLC has led to significant improvements in survival; however, recurrence remains common. New methods are needed to enhance anti-tumor immune responses and improve patient outcomes. Here, we present the first case of utilization of the Ethos OART platform to deliver multi-site pulsed hypofractionated radiotherapy in a patient with oligometastatic disease on the single arm prospective clinical trial SiCARIO (Split-Course Adaptive Radioimmunotherapy in Oligometastatic NSCLC, NCT05501665). A 67-year-old man with stage IV NSCLC with metastases to bilateral adrenal glands, retroperitoneum, and mesentery was prescribed treatment of 40 Gy in 5 fractions on SiCARIO in combination with SOC chemoimmunotherapy. A multi-target single isocenter approach was utilized to treat nine distinct targets in five total isocenters. Treatment plans were generated using an isotopic approach prioritizing organ at risk (OAR) constraints with the goal of minimum coverage of at least 30 Gy in 5 fractions. CBCT was acquired with each fraction to generate new targets and OAR contours based on anatomic changes with the patient on the treatment table. A comparison of an adapted plan to a base plan was performed online with a selection of superior plans based on target coverage and OAR constraints. The adapted plan was deemed superior for all but 1 fraction of a single isocenter for this patient. The discussion will focus primarily on the bilateral adrenal isocenter, where bulk tumor shrinkage of greater than 80% was observed in this patient with corresponding significant dosimetric benefits. This case demonstrates a potential clinical benefit of OART in multi-metastasis RT. Further data is needed to confirm the safety and efficacy of this approach. Enrollment is ongoing.

## Introduction

The addition of immunotherapy to the toolkit of the modern oncologist has dramatically changed the management of both localized and metastatic disease [1]. The introduction of immune checkpoint inhibitors now well over a decade ago has drastically changed our management of multiple malignancies, including melanoma, renal cell carcinoma, non-small cell lung cancer, cutaneous malignancies, and others. Benefit was initially found in the metastatic population for which these agents were first tested; however, these agents are increasingly being introduced in patients with earlier stage disease in the neoadjuvant and adjuvant settings with similar profound survival benefits. Use of immune checkpoint inhibitors (ICI) in patients with metastatic non-small cell lung cancer (NSCLC) has led to improvements in response rates and time to progression, although local and/or distant failure remains a near certainty [2,3]. This highlights a critical need for new strategies to promote more robust and durable responses to ICIs and improve patient outcomes.

An expanding body of evidence supports synergy between radiotherapy and immunotherapy. Radiation has multiple effects on tumor cells and the tumor microenvironment that may prime immunogenic cell death, including the presentation of damage-associated molecular patterns (DAMPs), activation of dendritic cells and increased antigen cross presentation, increased tumor-infiltrating lymphocytes (TILs), and upregulation of surface PD-L1 expression [4]. While efforts have been made to add ICIs to existing disease states treated with radiotherapy (i.e., the PACIFIC trial in stage III NSCLC), little has been done to change clinical radiation treatment paradigms to enhance the biologic interactions between radiotherapy and the immune system and better integrate these approaches into patients with oligometastatic and polymetastatic disease [5]. Emerging data supports the use of hypofractionated radiotherapy as the best approach to induce anti-tumor immune responses in combination with ICIs [6,7]. However, delivery of high dose per fraction radiotherapy, particularly in patients with multiple sites of disease, may be constricted by dose-limiting toxicities to organs at risk (OARs).

Online adaptive radiotherapy (OART) posits a solution by accounting for anatomic changes via real-time planning immediately prior to each daily fraction of radiotherapy [8,9]. Dosimetric benefit has been demonstrated in prior studies, which has even more significant implications for complex treatment plans due to larger treatment volumes and utilization of ablative radiation dosing, such as those for oligometastatic disease [10-12]. SiCARIO (Split-Course Adaptive Radioimmunotherapy in Oligometastatic NSCLC, NCT05501665) is a phase I/II clinical trial in patients with newly diagnosed stage IV non-small-cell lung cancer (NSCLC) with up to 10 sites of extracranial metastases using the Varian Ethos OART platform. Here, we present the case of a 67-year-old male with newly diagnosed stage IV NSCLC treated definitively with OART on SiCARIO, wherein pulsed high dose per fraction, anatomically adapted radiation was delivered concurrently with standard of care chemoimmunotherapy.

## Case presentation

Clinical presentation

**Figure 1:**
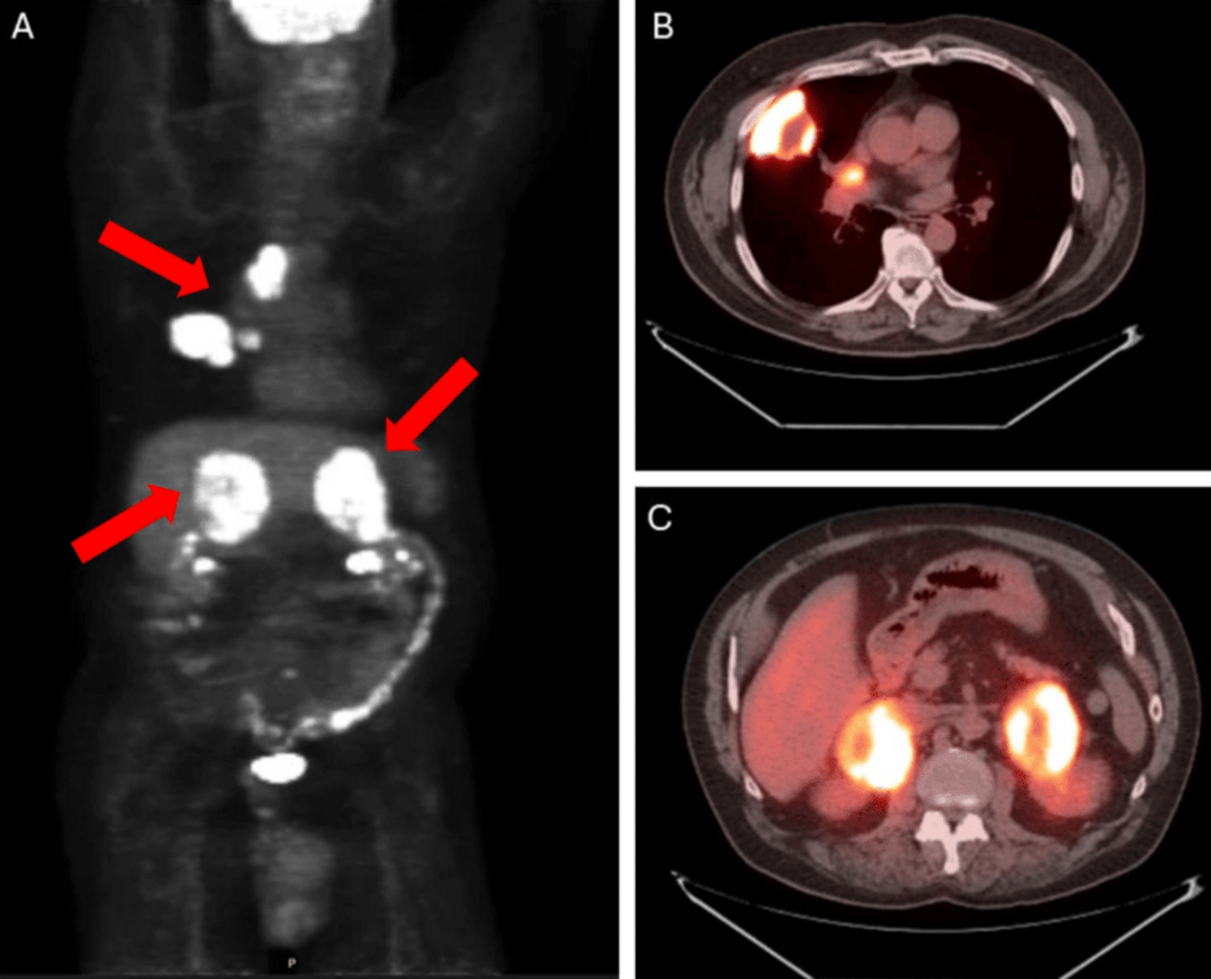
Initial staging PET-CT demonstrated multiple sites of FDG-avid disease. (A) Skull base to mid-thigh PET-CT images with arrows indicating thoracic and bilateral adrenal disease; (B) axial slice demonstrating lung mass and mediastinal disease; (C) axial slice demonstrating bulky bilateral adrenal metastases.

A 67-year-old male with a past medical history notable for obstructive sleep apnea, reflux, and osteoarthritis presented with sharp flank pain. Diagnostic imaging at that time was notable for adrenal nodules, but unfortunately, further investigation was delayed. Three months later, he underwent PET-CT, demonstrating a right upper lobe (RUL) lung mass (5.8 cm), mediastinal lymph node (4.5 cm), right hilar lymph node (2.8 cm), bilateral adrenal lesions (8 cm), a retroperitoneal nodule, and a mesenteric nodule, all FDG avid (Figure 1). 

The RUL mass was biopsied, demonstrating a SMARCA4-deficient undifferentiated tumor, TTF-1, Napsin A, p40 negative, PD-L1 <1%, suspicious for NSCLC. Brain MRI showed no intracranial abnormalities. He was thus determined to have stage IVB (cT3N2M1c) NSCLC. After discussion with the multi-disciplinary thoracic team, the patient was planned for treatment with 40 Gy in 5 fractions at all sites of disease with SOC carboplatin, paclitaxel, and pembrolizumab using the SiCARIO regimen (OART q3 weekly) on a Phase I/II clinical trial (NCT05501665).

Clinical trial details

**Figure 2:**
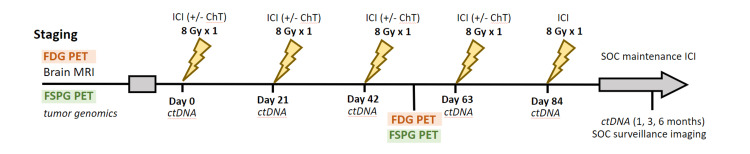
SiCARIO study schema. FDG PET: fluorodeoxyglucose positron emission tomography, MRI: magnetic resonance imaging, FSPG PET: (S)-4-(3-18F-fluoropropyl)-L-glutamic acid positron emission tomography, ICI: immune checkpoint inhibitor, ChT: chemotherapy, ctDNA: circulating tumor DNA, SOC: standard of care

NCT05501665 (SiCARIO) is a Phase I/II, single institution, open label clinical trial in patients with *de novo* stage IV NSCLC or locally advanced NSCLC (not eligible for SOC chemoradiation) with up to ten sites of extracranial metastases [13]. Patients with brain metastases which can be addressed with surgery and/or stereotactic radiosurgery are eligible. Patients receive RT to all sites of extracranial disease and thoracic primary in 8 Gy fractions every three weeks to a total dose of 40 Gy in 5 fractions concurrent with standard of care (SOC) (chemo)immunotherapy (Figure 2).

RT plans will be adapted using the Varian Ethos OART platform (Varian Medical Systems, Palo Alto, CA). Planned enrollment is 25 patients to observe an improvement in best overall response rate to 75% compared to historical controls. The primary objectives are to assess the safety, tolerability, and overall response rate (ORR) of the SiCARIO regimen combined with SOC systemic therapy. Secondary objectives include PFS, OS, and new metastasis-free survival. Exploratory objectives include serum biomarkers of response (ctDNA and immune profiling) and imaging biomarkers, including 18F-FDG and 18F-FSPG PET responses. This study will also serve as a practical evaluation of the Varian Ethos OART platform for the development of efficient clinical workflows integrating both anatomic and functional imaging data to deliver complex, multi-site RT plans. Enrollment is currently ongoing, with potential for future expansion pending initial outcomes.

Radiation treatment planning and delivery

**Figure 3:**
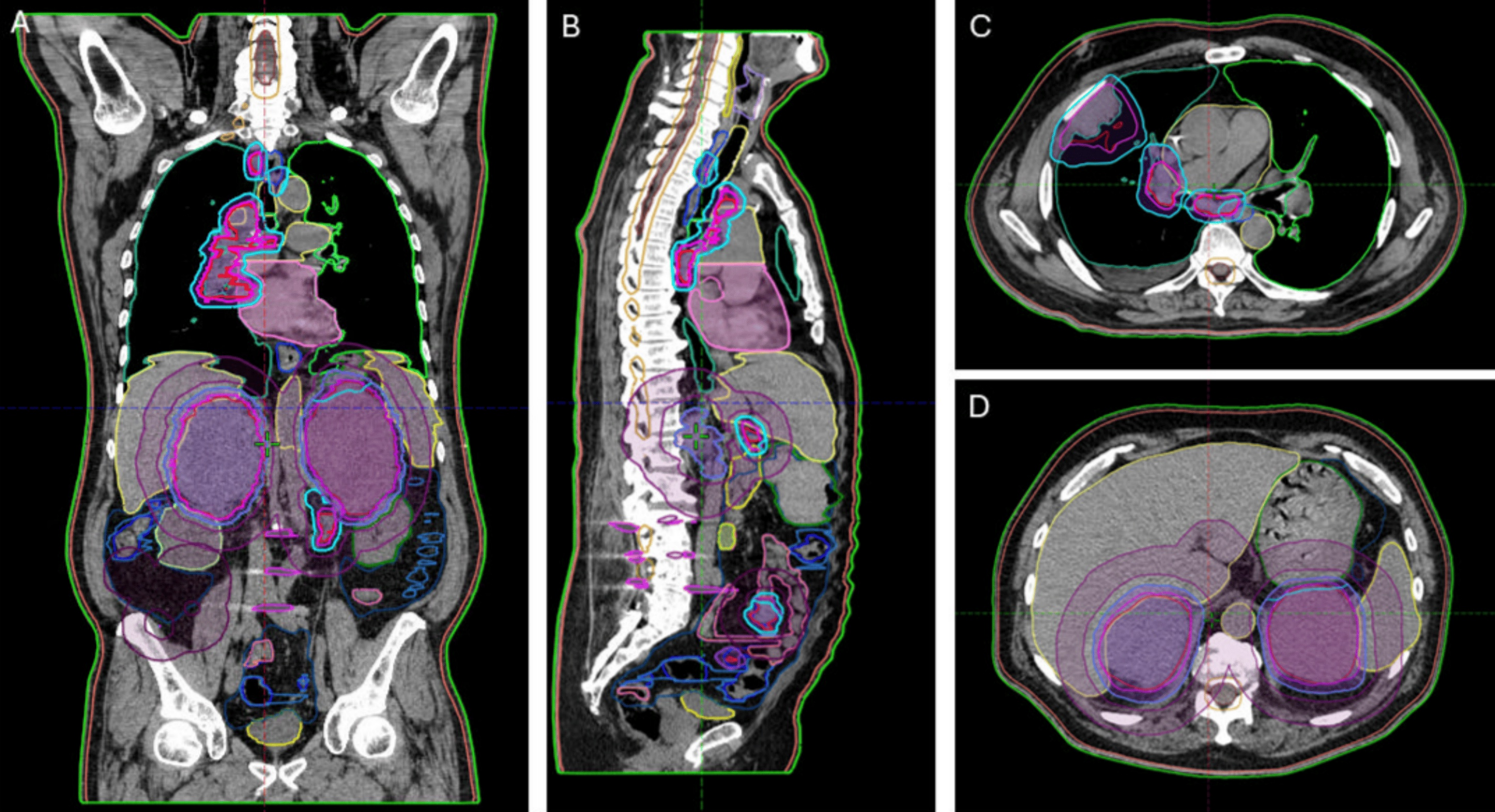
Representative images of multi-site target contours. (A) Representative coronal image, (B) Representative sagittal image, (C) Representative axial image demonstrating lung mass and mediastinal disease, (D) Representative axial image demonstrating bilateral adrenal disease.

The patient was simulated in a vacuum bag with a wingboard for immobilization. Four-dimensional CT was performed to assess respiratory motion. Gross tumor volumes (GTV) for all sites of disease were delineated using imaging from a simulation scan and fused diagnostic PET-CT and contrasted CT chest, abdomen, and pelvis. 4DCT was utilized to generate ITVs for structures with relevant intra-fraction respiratory motion. An institutional standard 5 mm isotropic expansion was added to generate PTVs (Figure 3).

Dose constraints and planning priorities were derived from institutional standards and relevant clinical trial protocols (Table 1).

**Table 1:** Target coverage goals and OAR constraints used in Ethos planning.

Structure	Goal	Priority
PTV_4000	D99%>30 Gy	1
PTV_4000	V35 > 90%	1
Small Bowel	D0.03cc < 32.5 Gy	1
Small Bowel	V32.5 < 0.03cc	1
Stomach	D0.03cc < 32.5 Gy	1
Stomach	V32.5 < 0.03cc	1
Large Bowel	D0.03cc < 34 Gy	1
Large Bowel	V34 < 0.03cc	1
Spinal canal	D0.03cc < 18 Gy	1
Both Kidneys	V20 < 110cc	2
Both Kidneys	V10 < 180cc	2
Both Kidneys	Dmean < 10 Gy	3
Both Kidneys	D0.03cc < 41.5 Gy	3
Liver	V21.5 < 550cc	2
Esophagus	D0.03cc < 34 Gy	1
Esophagus	D5cc < 31.5 Gy	2
Spinal canal + 0.5cm	D0.03cc < 22Gy	2
Both Lungs	V13.5 < 37%	2
Right Lung	V12.5 < 1500cc	3
Left Lung	V12.5 < 350cc	3
Heart	D0.03cc < 41.2 Gy	2
Heart	V41.2 < 0.03cc	2
Bronchial Tree	D0.03cc < 42 Gy	2
Bronchial Tree	V42 < 0.03cc	3
Great Vessels	D0.03cc < 41.5 Gy	2
Great Vessels	V41.5 < 0.03cc	3
Trachea	D0.03cc < 41.5 Gy	2
Trachea	V41.5 < 0.03cc	3

Plans were generated using an isotoxic approach, prioritizing meeting OAR constraints while seeking to deliver a minimum dose to target of at least 30 Gy in five fractions. Optimization structures were created as needed to meet these clinical goals. Multi-field static IMRT plans were utilized for all sites to minimize calculation time and patient on-table time.

Adapted plans were generated at each of the five treatment sessions based on changes in tumor and normal anatomy assessed utilizing on-table cone beam CT (CBCT). Using the Varian Ethos system, target and normal contours were automatically deformed based on relevant influencer structures. GTV and OARs were then manually edited at the discretion of the treating physician at the console. An online-adapted plan was generated by the treatment planning system, which was compared to the initial plan using both dose volume histogram (DVH) analysis and visual evaluation of 3D dose distribution. The superior plan was selected based on either or both of improved target coverage or reduced dose to OARs.

Dosimetric and clinical outcomes

**Figure 4:**
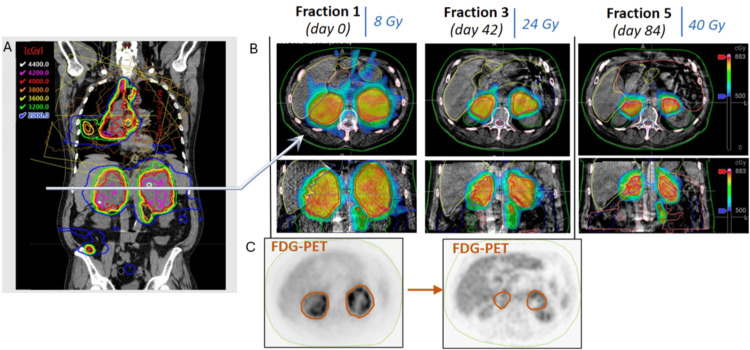
Base and adapted radiotherapy plans demonstrating interval response in bilateral adrenals. (A) Representative coronal image of the base radiotherapy plan for the multi-target plan. (B) Representative axial slices of bilateral adrenals demonstrating interval tumor shrinkage at fractions 3 and 5 from baseline. (C) Representative axial PET images demonstrating resolution of the FDG PET (fluorodeoxyglucose positron emission tomography) signal from baseline to prior to fraction 3.

The patient underwent five treatment sessions using the SiCARIO regimen at all sites of disease (one mesenteric lesion did receive only two fractions of the planned five fractions as it resolved during the course of treatment). The adapted plan was selected as superior in all but one fraction for a single isocenter during the treatment course due to the reduced dose to OARs. Patient reported no acute toxicities and noted improvement in cough and abdominal pain over a 12-week treatment course. For simplicity, further discussion will focus on the bilateral adrenal isocenter. Significant changes in tumor volume were observed in bilateral adrenal PTV targets (left adrenal: fraction 1: 571cc, fraction 2: 319cc, fraction 3: 197cc, fraction 4: 140cc, fraction 5: 91cc, right adrenal: fraction 1: 538cc, fraction 2: 338cc, fraction 3: 163cc, fraction 4: 106cc, fraction 5: 92cc) with 85% and 83% reductions in volume for left adrenal and right adrenal, respectively, over a 12-week treatment course. Additionally, FDG PET signal resolved from baseline to interim PET-CT prior to cycle 3 (Figure 4). 

**Figure 5:**
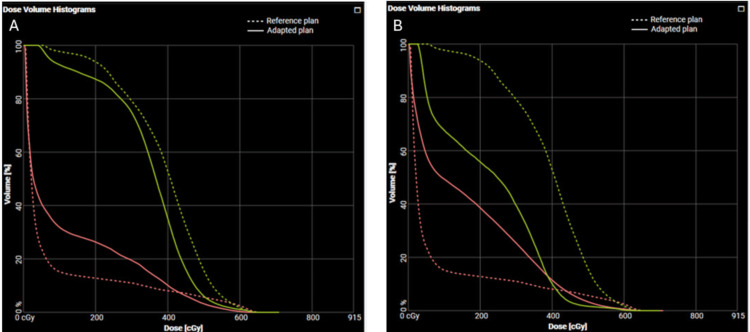
Dose volume histogram for fraction 3 and fraction 5 of bilateral adrenal RT plans. Comparison of reference (base) plan and adapted plan for bilateral adrenal isocenter. (A) Fraction 3 of treatment, demonstrating reduced Dmax to small bowel (peach) and stomach (yellow), solid: adapted plan, dashed: reference plan, (B) Fraction 5 of treatment, demonstrating reduced Dmax to small bowel (peach) and stomach (yellow); solid: adapted plan; dashed: reference plan. These fractions are representative of dosimetric benefits observed across all fractions, with approximately 20-25% reductions in Dmax to adjacent critical organs.

Clinically significant reductions in dose to critical OARs were observed using OART compared to the base plan while maintaining adequate target coverage. For example, maximum doses to the small bowel and stomach were reduced by 25% (867 cGy to 677 cGy) and 21% (854 cGy to 653 cGy), respectively, at fraction 3, and by 24% (885 cGy to 677 cGy) and 25% (872 cGy to 647 cGy) at fraction 5 (Figure 5).

Notably, doses on the base plan would have otherwise exceeded even our variation acceptable constraints for these critical OARs with OART facilitating delivery of clinically acceptable plans. 

Due to the complexity of targets and RT plans for the multi-target single isocenter plans utilized for this patient, patient time on the table was a concern. A total of nine targets were treated utilizing five separate isocenters, with treatment split over two separate days for each fraction. For the bilateral adrenal isocenter examined above (which represented the most complex isocenter based on target volumes), the time from initial CBCT to completion of dose delivery had a median time of 58 minutes, with approximately 15 minutes required for initial CBCT and editing of influencer structures, 25 minutes for further editing of OAR and target contours and adapted plan generation, 2 minutes for verification CBCT, and 16 minutes for RT delivery. For treatment of multiple isocenters on each day, the total on-table time for the patient was approximately 2 hours.

## Discussion

This case study suggests an impactful role for temporally fractionated OART in combination with immunotherapy in the aggressive management of newly diagnosed metastatic NSCLC. Historically, metastatic disease was primarily managed with chemotherapy. The dawn of immune checkpoint blockade therapy, along with a growing body of knowledge of immunobiology, has spurred the development of immune checkpoint inhibitors and other targeted therapies in the management of many cancers, including NSCLC [14,15]. In the last decade, immunotherapy has become a major player in lung cancer and even changed the standard of care for NSCLC since the impressive survival outcomes of the PACIFIC trial [16,17]. Furthermore, the addition of ablative radiotherapy in the oligometastatic setting has demonstrated a significant progression-free survival benefit [18]. Even so, prognosis remains poor, with a five-year overall survival of approximately 20%.

SiCARIO presents an opportunity to combine the benefits of immunotherapy and hypofractionated RT in patients with oligometastatic/polymetastatic disease. Moreover, utilization of OART provides a distinct dosimetric advantage and may reduce the toxicity of ablative treatments, as seen in this case study. OART facilitates an isotoxic approach to dose escalation for potential improvement in tumor control. Prior studies in NSCLC specifically have shown a decrease in lung V20Gy and heart V5 of around 5% while maintaining PTV coverage and even facilitating dose escalation using an adaptive approach [19]. OART also allows for significantly reduced setup margin, which is critical in mitigating normal tissue dose when treating several sites [20]. While promising, the clinical benefit of OART has not been prospectively demonstrated, and numerous studies are ongoing evaluating the approach [21,22].

Given the dramatic bulk tumor reduction in this patient, there is a potentially significant benefit to combining OART with pulsed fractionation, high-dose radiation, and immunotherapy. This could be related to immunostimulatory effects of hypofractionated RT promoting synergistic effects with ICI; however, a simple additive impact of ablative local treatment cannot be excluded. There is a paucity of data on high-dose, split-course radiotherapy, especially in concert with immunotherapy, which may serve as an ideal approach for this cohort. With the dramatic response seen in this patient, added to the dosimetric benefit of increased OAR sparing using OART, there is perhaps a role for pulsed fractionation OART in facilitating a durable tumor response while avoiding harm and improving quality of life in metastatic patients.

## Conclusions

Metastatic NSCLC continues to be associated with a poor prognosis despite advances in immunotherapy and dose-escalated radiotherapy. However, with proper patient selection, high-dose, split-course adapted radiotherapy with immunotherapy may harness the advantages that have been seen in ART, SBRT, and immunotherapy independently. In this report, we detail a case of the utilization of online adaptive radiotherapy (OART) to deliver pulsed hypofractionated radiation to multiple sites of disease concurrent with SOC systemic therapy in a patient with oligometastatic NSCLC. Use of CBCT-based OART allowed for structural adaptation to changing tumor and normal anatomy to deliver ablative doses of radiotherapy while decreasing cumulative dose to critical OARs, specifically small bowel and stomach for this patient. No CTCAE v5 grade 3 or higher hematologic or solid organ toxicities were observed. This novel, multimodal approach requires further exploration but presents a provocative option for patients with newly diagnosed metastatic NSCLC. Prospective evaluation of this approach is ongoing with further accrual on the SiCARIO Ph I/II trial. 
